# Posterior Elbow Dislocation with Brachial Artery Thrombosis Treated Non-surgically: A Case Report

**DOI:** 10.5704/MOJ.1711.008

**Published:** 2017-11

**Authors:** SM Lim, GG Chua, F Asrul, M Yazid

**Affiliations:** Department of Orthopaedics, Universiti Sains Malaysia, Kubang Kerian, Malaysia; ^*^Department of Orthopaedics, Hospital Tuanku Fauziah, Kangar, Malaysia

**Keywords:** elbow dislocation, brachial artery thrombosis

## Abstract

The brachial artery is rarely injured in closed posterior dislocation of the elbow, unlike the high rate of vascular injury seen after dislocation of the knee. Despite the anatomical proximity of the brachial artery to the elbow joint, most cases of brachial artery injury after dislocation of the elbow are related to an associated fracture, an open injury or high-energy trauma. A high index of suspicion should be maintained as well as a thorough neurovascular examination with regards this potentially disastrous complication. We describe an unusual case of complete thrombosis of the brachial artery presenting with a posterior elbow dislocation following a fall (low energy trauma) that was treated nonoperatively. At three months follow-up, patient had good circulation over the affected limb, no complaints of ischemic pain or cold intolerance, no signs of Volkmann’s ischemic contracture, and a range of motion that was comparable to the contralateral limb.

## Introduction

The brachial artery is rarely injured in closed elbow dislocation. The annual incidence of brachial artery injuries after closed elbow injuries ranges from 0.47% to 0.5% ^1^. The complications of brachial artery injury include gangrene and limb loss as well as long-term complications of limb ischemia including reduced range of motion, Volkmann’s contracture, and cold intolerance. We describe an unusual case of complete thrombosis of the brachial artery presenting with a posterior elbow dislocation following a fall, which was treated non-surgically. A high index of suspicion should be maintained with regards this potentially disastrous complication^2^.

## Case Report

**Fig. 1:**
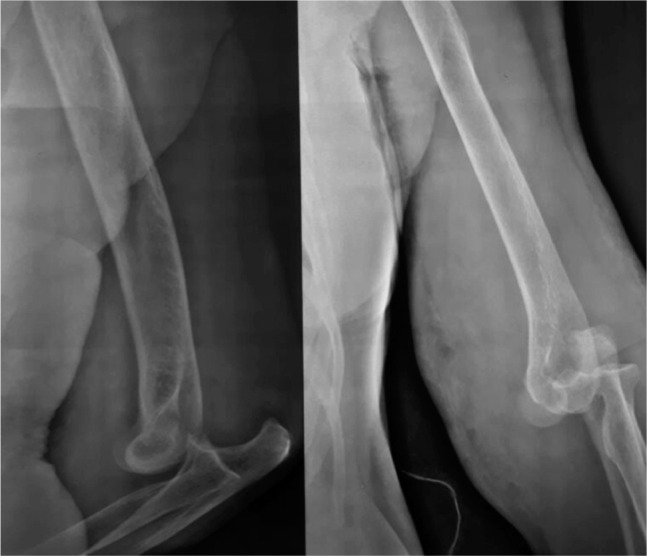
Pre-reduction elbow radiographs (Lateral and AP) showed a posterolateral dislocation of the left elbow. No fractures were noted.

**Fig. 2:**
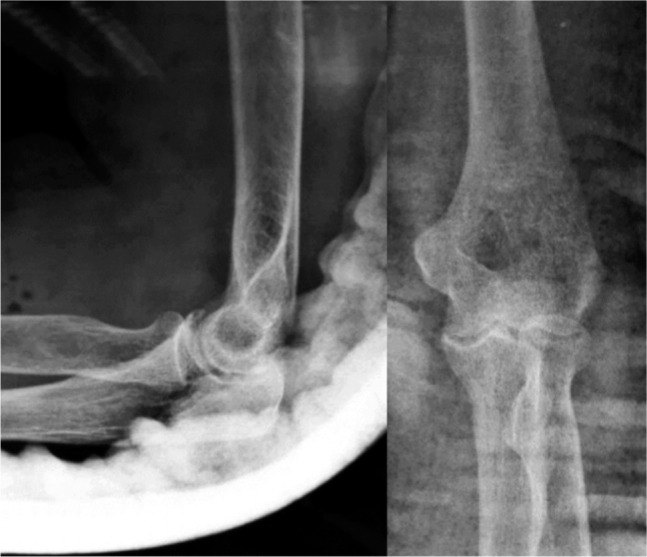
Post-reduction elbow radiographs (Lateral and AP) showing concentric reduction of the left elbow joint (upper limb in backslab).

A 76-year old woman had sustained a fall from standing position, resulting in dislocation of her left elbow. She presented at our centre within an hour of injury, with left elbow tenderness and deformity. The hand was warm and fingers were pink with capillary refill time (CRT) less than 2 seconds. Radial and ulnar pulses were not palpable but weak signal was detectable by Doppler ultrasound. Pulse oximetry over the fingers ranged from 80-90%. Sensation and gross motor function were intact. Radiographs (Fig. 1) showed a posterolateral dislocation of the elbow. Under monitored sedation, a closed reduction was performed and stability of joint was assessed, revealing no instability within the entire flexion extension arc; no valgus instability but slight varus laxity was noted. The elbow joint was immobilized with forearm in neutral position and 45 degrees flexion with a backslab, and was increased to 90 degrees flexion once swelling subsided. Post-reduction radiographs (Fig. 2) showed a concentric reduction of the elbow joint. After reduction, pulses were not palpable but detectable by Doppler, pulse oximetry over fingertips rose to 100%. Neurovascular status remained the same while patient was in the ward.

Four days later, she was re-examined and it was noted that the distal pulses were still not detectable by Doppler device, even though fingers were pink with CRT <2 seconds. A CT Angiogram revealed abrupt termination of contrast in the brachial artery about 3.7cm from elbow joint, with the non-visualized segment measuring 4cm, with no leakage of contrast. Reconstitution of the brachial artery was noted just distal to the elbow joint. There was very faint contrast opacification of the radial and ulnar arteries. CT angiogram suggested possible thrombosis of brachial artery. After consultation with a vascular surgeon, she was planned for non-surgical management. She was started on subcutaneous enoxaparin which was given for a week, followed by lifelong Aspirin 150mg OD. The patient was discharged from ward ten days post trauma.

At one month follow up, the fingers were pink with CRT <2 seconds, sensation and power intact. Distal pulses were still not palpable. Slab was removed; elbow range of motion was 15 to 100 degrees.

**Fig. 3:**
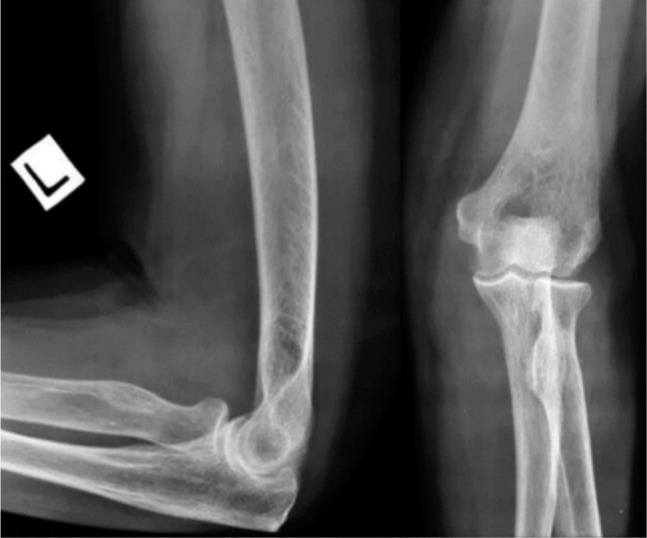
Radiographs of elbow (Lateral and AP) 3 months post trauma showing concentric left elbow joint.

At three months follow-up, she had no complaints of ischemic pain or cold intolerance. Circulation appeared good, distal pulses were palpable although slightly weaker than the contralateral side; range of motion was comparable to contralateral limb with no valgus or varus laxity. There were no signs of Volkmann’s ischemic contracture. Radiographs revealed concentric reduction (Fig. 3).

## Discussion

The elbow joint has multiple arterial anastomoses around it, both extraosseous and intraosseous. The extraosseous circulation is organized into three arcades situated medially, laterally and posteriorly. The intraosseous blood supply is derived from the feeding perforating vessels of the extraosseous arcades^3^.

The brachial artery is more vulnerable to injury at its distal end, because this portion may be trapped between the rigid bicipital aponeurosis and the dislocated bony structures, particularly the distal part of the humerus^4^.

There should be a high index of suspicion for brachial artery injury when a patient presents with blunt trauma from a fall onto the elbow, along with signs suggesting fracture or dislocation. Overt signs of limb ischemia are often not present, due to the rich collateral supply of the upper limb. Patients present with subtle signs such as palpable but diminished pulses, decreased capillary refill and reduced pulse oximetry readings. Such upper limbs are termed “pink pulseless hands” by Brahmamdam^5^.

The gold standard for diagnosis of arterial injury is CT angiography. However, some advocates of Doppler ultrasonography cite advantages that include convenience of execution at bedside, as well as it being less invasive; while others like Marcheix *et al* do not recommended it, reasoning that it is operator-dependant, and difficult to perform on an injured elbow^4^.

The patterns of brachial artery injury in elbow dislocations which had been reported in literature include thrombosis or intimal flaps at the injury site, or complete arterial transection^5^. While a limb with frank ischemia warrants urgent exploration and repair or grafting, the management of pink, pulseless hands is less clearly defined. As there has been no reported case of limb loss secondary to ischemia from an injury to the brachial artery associated with a closed dislocation, many controversies arise regarding the best method to treat these injuries, be it through observation, ligation, direct repair or vein grafting^1^. Observation alone has been reported successful in several cases of pink, pulseless hands. However, complications such as cold intolerance and intermittent claudication of the forearm and hand were noted.

Our patient had a simple, closed posterolateral elbow dislocation that had occurred after low energy trauma, which resulted in thrombosed but intact brachial artery. Other than undetected radial and ulna pulses, she had an otherwise clinically well perfused extremity. This patient was treated non-operatively for the vascular and ligamentous injury, and the result was satisfactory, with good clinical and functional outcome.

## Conclusion

Non-surgical treatment may be considered for certain vascular injury cases of the elbow, when there is a simple dislocation with brachial artery injury, but otherwise no signs of circulatory compromise. Complications associated with vascular repair such as infection, graft failure, compartment syndrome, subsequent surgeries and destruction of vital collaterals through surgical approach may be averted. In addition, considering the seemingly innocuous presentation of a pink limb with diminished radial and ulnar artery pulsations, signs of vascular injuries in a case of elbow dislocation should be actively sought out.
